# Supplementary material for: Functional Gene Clusters in Global Pathogenesis of Clear Cell Carcinoma of the Ovary Discovered by Integrated Analysis of Transcriptomes
Source: Int J Environ Res Public Health. 2020 Jun 2;17(11):3951. doi: 10.3390/ijerph17113951 (PMC7312065; doi:10.3390/ijerph17113951)
Supplement: Supplementary file 1 [file ijerph-17-03951-s001.pdf]

Supplementary Materials

Table S1. Sample number and mean of gene set regularity index for each group.

Case Number	Organism	Subtype	Microarry. Plateform	GSE.Series	Accession
1	Homo sapiens	Clear cell	GPL570	GSE44104	GSM1078980
2	Homo sapiens	Clear cell	GPL570	GSE44104	GSM1078979
3	Homo sapiens	Clear cell	GPL570	GSE44104	GSM1078977
4	Homo sapiens	Clear cell	GPL570	GSE44104	GSM1078976
5	Homo sapiens	Clear cell	GPL570	GSE44104	GSM1078974
6	Homo sapiens	Clear cell	GPL96	GSE6008	GSM139379
7	Homo sapiens	Clear cell	GPL570	GSE44104	GSM1078981
8	Homo sapiens	Clear cell	GPL570	GSE44104	GSM1078978
9	Homo sapiens	Clear cell	GPL570	GSE44104	GSM1078975
10	Homo sapiens	Clear cell	GPL570	GSE44104	GSM1078973
11	Homo sapiens	Clear cell	GPL570	GSE44104	GSM1078972
12	Homo sapiens	Clear cell	GPL570	GSE20565	GSM516756
13	Homo sapiens	Clear cell	GPL570	GSE26193	GSM642995
14	Homo sapiens	Clear cell	GPL570	GSE44104	GSM1078983
15	Homo sapiens	Clear cell	GPL570	GSE44104	GSM1078982
16	Homo sapiens	Clear cell	GPL96	GSE6008	GSM139378
17	Homo sapiens	Clear cell	GPL570	GSE20565	GSM516785
18	Homo sapiens	Clear cell	GPL570	GSE20565	GSM516743
19	Homo sapiens	Clear cell	GPL570	GSE55512	GSM1338409
20	Homo sapiens	Clear cell	GPL570	GSE26193	GSM643018
21	Homo sapiens	Clear cell	GPL570	GSE26193	GSM642986
22	Homo sapiens	Clear cell	GPL7264	GSE51088	GSM1238214
23	Homo sapiens	Clear cell	GPL96	GSE6008	GSM139383
24	Homo sapiens	Clear cell	GPL96	GSE6008	GSM139382
25	Homo sapiens	Clear cell	GPL96	GSE6008	GSM139380
26	Homo sapiens	Clear cell	GPL96	GSE14764	GSM368686
27	Homo sapiens	Clear cell	GPL96	GSE14764	GSM368676
28	Homo sapiens	Clear cell	GPL570	GSE20565	GSM516721
29	Homo sapiens	Clear cell	GPL570	GSE55512	GSM1338407
30	Homo sapiens	Clear cell	GPL570	GSE26193	GSM642971
31	Homo sapiens	Clear cell	GPL7264	GSE51088	GSM1238258
32	Homo sapiens	Clear cell	GPL96	GSE6008	GSM139384
33	Homo sapiens	Clear cell	GPL96	GSE14764	GSM368684
34	Homo sapiens	Clear cell	GPL570	GSE20565	GSM516764
35	Homo sapiens	Clear cell	GPL570	GSE26193	GSM643001
36	Homo sapiens	Clear cell	GPL96	GSE6008	GSM139381
37	Homo sapiens	Clear cell	GPL570	GSE20565	GSM516711
38	Homo sapiens	Clear cell	GPL570	GSE26193	GSM642962
39	Homo sapiens	Clear cell	GPL570	GSE30161	GSM746874
40	Homo sapiens	Clear cell	GPL570	GSE30161	GSM746865
41	Homo sapiens	Clear cell	GPL570	GSE30161	GSM746883
42	Homo sapiens	Clear cell	GPL570	GSE30161	GSM746870
43	Homo sapiens	Clear cell	GPL7264	GSE51088	GSM1238252
44	Homo sapiens	Clear cell	GPL96	GSE6008	GSM139377
45	Homo sapiens	Clear cell	GPL570	GSE63885	GSM1559399

46	Homo sapiens	Clear cell	GPL570	GSE63885	GSM1559398
47	Homo sapiens	Clear cell	GPL570	GSE63885	GSM1559397
48	Homo sapiens	Clear cell	GPL570	GSE63885	GSM1559396
49	Homo sapiens	Clear cell	GPL570	GSE63885	GSM1559395
50	Homo sapiens	Clear cell	GPL570	GSE63885	GSM1559394
51	Homo sapiens	Clear cell	GPL570	GSE63885	GSM1559393
52	Homo sapiens	Clear cell	GPL570	GSE63885	GSM1559392
53	Homo sapiens	Clear cell	GPL570	GSE63885	GSM1559391
54	Homo sapiens	Clear cell	GPL570	GSE29450	GSM729043
55	Homo sapiens	Clear cell	GPL570	GSE29450	GSM729042
56	Homo sapiens	Clear cell	GPL570	GSE29450	GSM729041
57	Homo sapiens	Clear cell	GPL570	GSE29450	GSM729040
58	Homo sapiens	Clear cell	GPL570	GSE29450	GSM729039
59	Homo sapiens	Clear cell	GPL570	GSE29450	GSM729038
60	Homo sapiens	Clear cell	GPL570	GSE29450	GSM729037
61	Homo sapiens	Clear cell	GPL570	GSE29450	GSM729036
62	Homo sapiens	Clear cell	GPL570	GSE29450	GSM729035
63	Homo sapiens	Clear cell	GPL570	GSE29450	GSM729034
64	Homo sapiens	Clear cell	GPL6244	GSE54807 GSE54809	GSM1324310
65	Homo sapiens	Clear cell	GPL6244	GSE54807 GSE54809	GSM1324307
66	Homo sapiens	Clear cell	GPL6244	GSE54807 GSE54809	GSM1324305
67	Homo sapiens	Clear cell	GPL6244	GSE54807 GSE54809	GSM1324299
68	Homo sapiens	Clear cell	GPL6244	GSE54807 GSE54809	GSM1324298
69	Homo sapiens	Clear cell	GPL6244	GSE54807 GSE54809	GSM1324295
70	Homo sapiens	Clear cell	GPL6244	GSE54807 GSE54809	GSM1324293
71	Homo sapiens	Clear cell	GPL6244	GSE54807 GSE54809	GSM1324292
72	Homo sapiens	Clear cell	GPL6244	GSE54807 GSE54809	GSM1324291
73	Homo sapiens	Clear cell	GPL6244	GSE54807 GSE54809	GSM1324290
74	Homo sapiens	Clear cell	GPL6244	GSE54807 GSE54809	GSM1324288
75	Homo sapiens	Clear cell	GPL6244	GSE54807 GSE54809	GSM1324287
76	Homo sapiens	Clear cell	GPL6947	GSE16570 GSE16574	GSM416684
77	Homo sapiens	Clear cell	GPL6947	GSE16570 GSE16574	GSM416683
78	Homo sapiens	Clear cell	GPL6947	GSE16570 GSE16574	GSM416682
79	Homo sapiens	Clear cell	GPL6947	GSE16570 GSE16574	GSM416681
80	Homo sapiens	Clear cell	GPL6947	GSE16570 GSE16574	GSM416680
1	Homo sapiens	normal	GPL570	GSE12034 Dataset	GSM304262
2	Homo sapiens	normal	GPL570	GSE12034 Dataset	GSM304261
3	Homo sapiens	normal	GPL570	GSE12034 Dataset	GSM304260
4	Homo sapiens	normal	GPL570	GSE34526	GSM850529
5	Homo sapiens	normal	GPL570	GSE34527	GSM850528
6	Homo sapiens	normal	GPL570	GSE34528	GSM850527
7	Homo sapiens	normal	GPL570	GSE36668	GSM898308
8	Homo sapiens	normal	GPL570	GSE36668	GSM898307
9	Homo sapiens	normal	GPL570	GSE36668	GSM898306
10	Homo sapiens	normal	GPL570	GSE36668	GSM898305
11	Homo sapiens	normal	GPL570	GSE14407	GSM359983
12	Homo sapiens	normal	GPL570	GSE14407	GSM359982
13	Homo sapiens	normal	GPL570	GSE14407	GSM359981
14	Homo sapiens	normal	GPL570	GSE14407	GSM359980
15	Homo sapiens	normal	GPL570	GSE14407	GSM359979
16	Homo sapiens	normal	GPL570	GSE14407	GSM359978

17	Homo sapiens	normal	GPL570	GSE14407	GSM359977
18	Homo sapiens	normal	GPL570	GSE14407	GSM359976
19	Homo sapiens	normal	GPL570	GSE14407	GSM359975
20	Homo sapiens	normal	GPL570	GSE14407	GSM359974
21	Homo sapiens	normal	GPL570	GSE14407	GSM359973
22	Homo sapiens	normal	GPL570	GSE14407	GSM359972
23	Homo sapiens	normal	GPL570	GSE19383	GSM481013
24	Homo sapiens	normal	GPL570	GSE19383	GSM481012
25	Homo sapiens	normal	GPL570	GSE19383	GSM481011
26	Homo sapiens	normal	GPL570	GSE19383	GSM481010
27	Homo sapiens	normal	GPL570	GSE19383	GSM481009
28	Homo sapiens	normal	GPL570	GSE19383	GSM481008
29	Homo sapiens	normal	GPL570	GSE18520 GSE18521	GSM462652
30	Homo sapiens	normal	GPL570	GSE18520 GSE18521	GSM462651
31	Homo sapiens	normal	GPL570	GSE18520 GSE18521	GSM462650
32	Homo sapiens	normal	GPL570	GSE18520 GSE18521	GSM462649
33	Homo sapiens	normal	GPL570	GSE18520 GSE18521	GSM462648
34	Homo sapiens	normal	GPL570	GSE18520 GSE18521	GSM462647
35	Homo sapiens	normal	GPL570	GSE18520 GSE18521	GSM462646
36	Homo sapiens	normal	GPL570	GSE18520 GSE18521	GSM462645
37	Homo sapiens	normal	GPL570	GSE18520 GSE18521	GSM462644
38	Homo sapiens	normal	GPL570	GSE18520 GSE18521	GSM462643
39	Homo sapiens	normal	GPL570	GSE7307	GSM176318
40	Homo sapiens	normal	GPL570	GSE7307	GSM176237
41	Homo sapiens	normal	GPL570	GSE7307	GSM176136
42	Homo sapiens	normal	GPL570	GSE7307	GSM176131
43	Homo sapiens	normal	GPL570	GSE7307	GSM175789
44	Homo sapiens	normal	GPL570	GSE23391 GSE23392	GSM573685
45	Homo sapiens	normal	GPL570	GSE23391 GSE23392	GSM573684
46	Homo sapiens	normal	GPL570	GSE23391 GSE23392	GSM573683
47	Homo sapiens	normal	GPL570	GSE23391 GSE23392	GSM573682
48	Homo sapiens	normal	GPL570	GSE23391 GSE23392	GSM573681
49	Homo sapiens	normal	GPL570	GSE52037 GSE52460	GSM1257889
50	Homo sapiens	normal	GPL570	GSE52037 GSE52460	GSM1257888
51	Homo sapiens	normal	GPL570	GSE52037 GSE52460	GSM1257887
52	Homo sapiens	normal	GPL570	GSE52037 GSE52460	GSM1257886
53	Homo sapiens	normal	GPL570	GSE52037 GSE52460	GSM1257885
54	Homo sapiens	normal	GPL570	GSE52037 GSE52460	GSM1257884
55	Homo sapiens	normal	GPL570	GSE52037 GSE52460	GSM1257883
56	Homo sapiens	normal	GPL570	GSE52037 GSE52460	GSM1257882
57	Homo sapiens	normal	GPL570	GSE52037 GSE52460	GSM1257881
58	Homo sapiens	normal	GPL570	GSE52037 GSE52460	GSM1257880
59	Homo sapiens	normal	GPL570	GSE27651	GSM372263
60	Homo sapiens	normal	GPL570	GSE27651	GSM372262
61	Homo sapiens	normal	GPL570	GSE27651	GSM372261
62	Homo sapiens	normal	GPL570	GSE27651	GSM372260
63	Homo sapiens	normal	GPL570	GSE27651	GSM372259
64	Homo sapiens	normal	GPL570	GSE27651	GSM372258
65	Homo sapiens	normal	GPL570	GSE25427 GSE25429	GSM624163
66	Homo sapiens	normal	GPL570	GSE25427 GSE25429	GSM624162
67	Homo sapiens	normal	GPL570	GSE25427 GSE25429	GSM624146

68	Homo sapiens	normal	GPL570	GSE25427 GSE25429	GSM624145
69	Homo sapiens	normal	GPL570	GSE14001	GSM351320
70	Homo sapiens	normal	GPL570	GSE14001	GSM351318
71	Homo sapiens	normal	GPL570	GSE40266	GSM989499
72	Homo sapiens	normal	GPL570	GSE40266	GSM989496
73	Homo sapiens	normal	GPL570	GSE40266	GSM989494
74	Homo sapiens	normal	GPL570	GSE40266	GSM989492
75	Homo sapiens	normal	GPL570	GSE40266	GSM989490
76	Homo sapiens	normal	GPL570	GSE40266	GSM989488
77	Homo sapiens	normal	GPL570	GSE40266	GSM989485
78	Homo sapiens	normal	GPL570	GSE40266	GSM989483
79	Homo sapiens	normal	GPL570	GSE40266	GSM989481
80	Homo sapiens	normal	GPL570	GSE15578	GSM389794
81	Homo sapiens	normal	GPL570	GSE15578	GSM389791
82	Homo sapiens	normal	GPL570	GSE15578	GSM389790
83	Homo sapiens	normal	GPL570	GSE15578	GSM389789
84	Homo sapiens	normal	GPL570	GSE15578	GSM389783
85	Homo sapiens	normal	GPL570	GSE15578	GSM389782
86	Homo sapiens	normal	GPL570	GSE29450	GSM729053
87	Homo sapiens	normal	GPL570	GSE29450	GSM729052
88	Homo sapiens	normal	GPL570	GSE29450	GSM729051
89	Homo sapiens	normal	GPL570	GSE29450	GSM729050
90	Homo sapiens	normal	GPL570	GSE29450	GSM729049
91	Homo sapiens	normal	GPL570	GSE29450	GSM729048
92	Homo sapiens	normal	GPL570	GSE29450	GSM729047
93	Homo sapiens	normal	GPL570	GSE29450	GSM729046
94	Homo sapiens	normal	GPL570	GSE29450	GSM729045
95	Homo sapiens	normal	GPL570	GSE29450	GSM729044
96	Homo sapiens	normal	GPL6947	GSE16570 GSE16574	GSM416674
97	Homo sapiens	normal	GPL6947	GSE16570 GSE16574	GSM416673
98	Homo sapiens	normal	GPL6947	GSE16570 GSE16574	GSM416672
99	Homo sapiens	normal	GPL6947	GSE16570 GSE16574	GSM416671
100	Homo sapiens	normal	GPL6947	GSE16570 GSE16574	GSM416670
